# The Journal *Investigación y Educación en Enfermería* is Indexed in PubMed central

**DOI:** 10.17533/udea.iee.v39n1e01

**Published:** 2021-03-05

**Authors:** María de los Ángeles Rodríguez-Gázquez

**Affiliations:** 1 RN, Ph.D. Editor of Investigación y Educación en Enfermería, Universidad de Antioquia, Medellín (Colombia) Email: revistaiee@udea.edu.co Universidad de Antioquia Universidad de Antioquia Medellín Colombia revistaiee@udea.edu.co

As Editor, it is a joy to share with you the great news that the journal *Investigación y Educación en Enfermería* was accepted for indexation in PubMed Central (PMC), which is the world’s largest repository of knowledge on health sciences. PubMed Central has close to seven million articles in full text and in Open Access that can be recovered through the PubMed search engine.

Currently, PMC archives over 3018 journals accepted after demanding scientific and technical evaluations. Being included in this repository is a recognition distinction of the quality of our publication with the global academic community, with 22 nursing journals sharing this honor globally and only two of these are Latin American.

The journal *Investigación y Educación en Enfermería* has also been part of MEDLINE since 2014, a fact that marked a milestone on the excellence of our journal. We are convinced that with the new PMC indexation, visibility will increase further of the articles published in our journal, thus, improving the dissemination of the knowledge produced in nursing research.

Lastly, I wish to thank the authors, reviewers, members of the editorial committee, and, most specially, I thank our readers for their loyalty throughout these years. We count on your perseverance and enthusiasm to make this the best medium.

I share with you the congratulatory messages from the members of the Editorial Committee of the Journal Investigación y Educación en Enfermería upon its indexation in PubMed Central:

Carmen de la Cuesta y Benjumea. Universidad de Alicante, Spain: "Congratulations, María de los Ángeles, and thank you for sharing the link. It provides phenomenal visibility to the journal and to the work of all the authors".

Isabel Amélia Costa Mendes. Universidade de São Paulo, School of Nursing of Ribeirão Preto, Brazil: "Dear, María de los Ángeles, what great news! Congratulations to you and your entire team. I know how much effort you have invested throughout this trajectory and, with it, much more success will come to improve and give further value to Nursing. Receive my compliments as nurse, researcher, member of the editorial committee of this important and outstanding journal, Investigación y Educación Enfermería. I also record my effusive greetings as coordinator of the Iberian-American Network of Scientific Editing in Nursing -RedEDIT-".

Cristina García Vivar. Universidad Pública de Navarra, Spain: "It is a success that the journal is indexed in PMC, given that as you state it, this is a badge of recognition of the journal’s quality. Undoubtedly, this success is due to the steadfast work you have performed as editor, as well as to the authors who have submitted their articles for publication in the journal".

Rafael Fernández Castillo, Universidad de Granada, Spain: "I am proud to be with you and to be part of the editorial staff".

Martha Lucía Vásquez Truissi. Universidad del Valle, Colombia: Congratulations! This accomplishment is the product of effort, perseverance, and discipline; characteristics that have always distinguished the Journal.

Neusa Collet. Universidade Federal da Paraíba, Brazil: "What great news! Congratulations for the work done with great judgment and scientific criteria. It is a giant leap for the Journal, much more success will come!"

Manuel Alves Rodrigues. Escola Superior de Enfermagem de Coimbra, Portugal: "Good work! Greetings also to the entire editorial staff".

José Rafael González López. Universidad de Sevilla, Spain. "My most sincere congratulations for the recognition, the fruit of your excellent work and dedication as editor. A kiss and thank you very much again for having me on the Journal’s Editorial Committee".

R. Mauricio Barría P. Universidad Austral, Chile. "Esteemed, María de los Ángeles, thank you for sharing this great news. This, without a doubt, is another sign of recognition to the exhaustive and committed work carried out by the entire Journal staff under your management and leadership. I am proud to be part of the Journal and I express my commitment to continue working with all of you in the transition to reach and consolidate new achievements".

Gladys Eugenia Canaval Erazo. Universidad del Valle, Colombia. "Dear, María de los Ángeles: Receive my greetings for this achievement extended to all members of the staff. What pride for all who are involved with the Journal in one way or another".

Sonia Semenic. McGill University, Canada. “Congratulations on having the journal indexed in PubMed Central! It is a great testament to your hard work and expertise as an editor.”

